# Diagnostic Tests in the Prediction of Neonatal Outcome in Early Placental Fetal Growth Restriction

**DOI:** 10.3390/medicina59020406

**Published:** 2023-02-19

**Authors:** Vesna Mandić-Marković, Mirjana Bogavac, Željko Miković, Milan Panić, Dejan M. Pavlović, Jelena Mitrović, Milica Mandić

**Affiliations:** 1 Faculty of Medicine, University of Belgrade, Dr Subotica 8, 11000 Belgrade, Serbia; 2 Department of High-Risk Pregnancies, University Clinic for Gynecology and Obstetrics “Narodni Front”, Kraljice Natalije 62, 11000 Belgrade, Serbia; 3 Department of Obstetrics and Gynecology, Faculty of Medicine University of Novi Sad, Branimira Ćosića 37, 21000 Novi Sad, Serbia

**Keywords:** fetal growth restriction, preterm, diagnostic parameters, Doppler, fetal heart rate monitoring

## Abstract

*Background and Objectives*: Monitoring pregnancies with fetal growth restriction (FGR) presents a challenge, especially concerning the time of delivery in cases of early preterm pregnancies below 32 weeks. The aim of our study was to compare different diagnostic parameters in growth-restricted preterm neonates with and without morbidity/mortality and to determine sensitivity and specificity of diagnostic parameters for monitoring preterm pregnancies with early preterm fetal growth restriction below 32 weeks. *Materials and Methods*: Our clinical study evaluated 120 cases of early preterm deliveries, with gestational age ≤ 32 + 0 weeks, with prenatally diagnosed placental FGR. All the patients were divided into three groups of 40 cases each based on neonatal condition,: I—Neonates with morbidity/mortality (NMM); II—Neonates without morbidity with acidosis/asphyxia (NAA); III—Neonates without neonatal morbidity/acidosis/asphyxia (NWMAA). *Results*: Amniotic fluid index (AFI) was lower in NMM, while NWMAA had higher biophysical profile scores (BPS). UA PI was lower in NWMAA. NWMAA had higher MCA PI and CPR and fewer cases with CPR <5th percentile. NMM had higher DV PI, and more often had ductus venosus (DV) PI > 95th‰ or absent/reversed A wave, and pulsatile blood flow in umbilical vein (UV). The incidence of pathological fetal heart rate monitoring (FHRM) was higher in NMM and NAA, although the difference was not statistically significant. ROC calculated by defining a bad outcome as NMM and a good outcome as NAA and NWMAA showed the best sensitivity in DV PIi. ROC calculated by defined bad outcome in NMM and NAA and good outcome in NWMAA showed the best sensitivity in MCA PI. *Conclusions*: In early fetal growth restriction normal cerebral blood flow strongly predicts good outcomes, while pathological venous blood flow is associated with bad outcomes. In fetal growth restriction before 32 weeks, individualized expectant management remains the best option for the optimal timing of delivery.

## 1. Introduction

Fetal growth restriction (FGR) is defined as estimated or actual weight below the 10th percentile for gestational age and presents increased risk for perinatal morbidity and mortality [1,2]. FGR of placental origin is clinically the most relevant, as long-lasting malnutrition and hypoxemia might trigger hemodynamic changes resulting in activation of anaerobic metabolism and consequent neonatal morbidity/mortality [1,2]. Perinatal morbidity/mortality are additionally increased in early preterm pregnancies below 32 weeks [3]. FGR elective delivery reduces stillbirth but increases neonatal morbidity and mortality due to iatrogenic prematurity [4]. Survival in extremely premature FGR is minimal and almost always connected with severe morbidity and neurological sequels [5,6,7,8].

Monitoring pregnancies with early preterm FGR presents a challenge, especially when it comes to the time of delivery. The imperative of many studies is to find the optimal method for timely detection of fetal hypoxemia and asphyxia to plan elective delivery. Monitoring is carried out by ultrasound (US) and fetal heart rate monitoring (FHRM) [1,2]. FHRM is visually assessed for fetal heart rate (FHR) frequency, variability, accelerations, and decelerations. US monitoring implies biophysical profile score (BPS), amniotic fluid index (AFI) and color Doppler measurement of blood flow in umbilical, cerebral and venous circulation. If all methods show uniform findings, i.e., satisfactory, or unsatisfactory fetal condition, the decision about delivery is relatively easy, but if findings are different, the dilemma about preferable diagnostic parameter may arise, especially in early preterm pregnancies where the mother’s condition has been found to be satisfactory.

The aim of our study was to compare different diagnostic parameters in growth-restricted preterm neonates with and without morbidity/mortality and to determine sensitivity and specificity of diagnostic parameters for monitoring preterm pregnancies with early preterm fetal growth restriction below 32 weeks.

## 2. Materials and Methods

Observational clinical study was conducted at the University Clinic for Gynecology and Obstetrics “Narodni Front”, Belgrade. The study evaluated 120 cases of early preterm deliveries, with gestational ages ≤ 32 + 0 weeks, with prenatally diagnosed placental fetal growth restriction. All the pregnancies were single, with determined gestational age (by last menstrual period, confirmed by first trimester ultrasound), and without any congenital malformations, chromosomal abnormalities or congenital infections. All the patients were divided into three groups of 40 cases each based on neonatal condition: I—Neonates with morbidity/mortality (NMM); II—Neonates without morbidity with acidosis/asphyxia (NAA); and III—Neonates without neonatal morbidity/acidosis/asphyxia (NWMAA). The study aimed to form the groups with identical number of patients. We recruited 90 patients after hospital admittance, and followed them prospectively. After delivery and neonatal follow-up, each patient was classified into the appropriate group. There were 32 patients in the NMM group, 27 patients in the NAA group and 21 patients in the NWMAA group. The groups were fulfilled retrospectively with 30 patients, treated and delivered in the same hospital, who were selected after delivery when neonatal outcome was evident. All the patients had signed an informed consent form. The diagnosis of FGR was made during pregnancy by estimated fetal weight using Hadlock’s formula and confirmed after delivery when neonatal body weight was lower than the 10th percentile for our population [9,10]. Asymmetric FGR had been diagnosed during pregnancy using fetal growth parameters (HC/AC, FL/BPD and FL/AC), and after birth by measuring ponderal index (birth weight/crown-heel length 3 × 100), and skin-fold measurements 12 h after delivery on the left side of the body, using a Holtain caliper at the triceps and subscapular sites., FGR was diagnosed when these measurements were lower than the 10th percentile according to our population and the Rodriguez tables [11]. Placental FGR was diagnosed by measurement of uterine artery blood flow and defined as a mean PI and/or mean RI higher than the 95th percentile and by the presence of notching [12].

Each patient underwent ultrasound examination and FHRM at a frequency determined by the managing obstetrician based on gestational age and estimated fetal wellbeing. Amniotic fluid index (AFI) and biophysical profile score (BPS) were expressed in numeric values according to the method of Manning [13,14]. Umbilical artery (UA) blood flow was measured in a free loop and expressed as either (a) PI, or (b) descriptive UA blood flow (PI ≤ 95th percentile; PI > 95th percentile or absent/reversed end-diastolic blood flow—AREDV) [15,16]. Cerebral blood flow was measured at the proximal part of the middle cerebral artery (MCA) and expressed as either (a) PI, or (b) cerebral–placental ratio—CPR ($\mathrm{CRP}=\frac{\mathrm{PI}\;\mathrm{ACM}}{\mathrm{PI}\;A\;\mathrm{Umb}}$), or (c) descriptive—ΔCPR (CPR ≥ 5th percentile or CPR < 5th percentile) [16,17]. Venous blood flow was observed in the ductus venosus and umbilical vein. Ductus venosus (DV) blood flow was measured in the transverse abdominal section and expressed as either (a) Pi, or (b) descriptive DV blood flow (Pi ≤ 95th percentile; Pi > 95th percentile or absent/reversed A wave) [18]. Umbilical vein (UV) blood flow was expressed as laminar or pulsatile. FHRM was expressed as (a) normal/silent, or (b) spontaneous decelerations during non-stress test (NST)/positive contraction stress test (CST).

The indications for delivery were the presence of fetal compromise or worsening of maternal wellbeing. Indications of fetal compromise included Doppler changes (absent/reverse end diastolic flow in umbilical artery and/or absent/reverse A wave in ductus venosus), BPS ≤ 4, and pathological FHRM monitoring (decelerations and/or positive contraction stress test). Maternal indications for delivery included severe pre-eclampsia, superimposed pre-eclampsia, and HELLP (Hemolysis, elevated liver enzymes, low platelet) syndrome. When possible, fetal lung maturation was carried out using 24 mg of Dexamethason for 48 h and neuroprotective therapy for at least 12 h with MgSO_4_, ending at least 6 h before delivery.

Next, the birth cord arterial acid–base status was determined. Acidosis was diagnosed when pH ≤ 7.15, and base excess (BE) >12. Neonatal asphyxia was diagnosed if the 5’min Apgar score was <5. We registered neonatal morbidity and mortality. Neonatal morbidity was diagnosed based on CNS lesions (intraventricular hemorrhage, periventricular hemorrhage, leucomalatia, convulsions, and hypoxic-ischemic encephalopathy), pneumonia, severe RDS with need for mechanical ventilation, sepsis (positive culture with antibiotic treatment), and necrotizing enterocolitis (NEC). Neonates diagnosed with any of the above listed morbidities, or cases of neonatal death, were listed in the NMM group. Neonates who had acidosis and/or asphyxia after birth, but who did not have any of the listed morbidities, were listed in the NAA group. Neonates who had neither acidosis/asphyxia, nor any of the listed morbidities, were listed in the NWMAA group. In the NMM group there were eleven neonatal deaths, eight neonates exhibited RDS and sepsis, four exhibited RDS and pneumonia, one exhibited RDS, sepsis and pneumonia, five exhibited RDS and CNS lesions, two exhibited RDS, CNS lesions and sepsis, three exhibited RDS, two exhibited sepsis and CNS lesions, five exhibited CNS lesions, and one had sepsis and NEC. In the NAA group, 21 neonates had asphyxia, six had acidosis, and thirteen had both asphyxia and acidosis.

We also registered maternal age, parity, presence of pre-eclampsia, gestational age at birth, corticosteroid therapy, neuroprotection, indication for delivery (maternal or fetal), delivery mode (Cesarean section (CS) or vaginal), neonatal body weight, 5 min Apgar score, umbilical artery pH, base excess, presence of asphyxia, neonatal morbidity (intraventricular hemorrhage, periventricular hemorrhage, leucomalatia, convulsions, and hypoxic–ischemic encephalopathy, pneumonia, sepsis, severe RDS, and NEC), and neonatal mortality.

Statistical processing and analysis were performed in the statistical package SPSS version 24 (Statistical Package for the Social Sciences, IBM SPSS Statistics software version 24.0 for Windows, Armonk, NY, USA). Statistical analysis was performed by calculating means and standard deviations, χ^2^ (chi-square test), ANOVA test, and independent Samples *t* Test. Comparison was made between the groups. Receiver operating (ROC) was carried out to determine the sensitivity and specificity of each diagnostic parameter comparing good and bad neonatal outcomes. ROC was carried out with two criteria of good and bad outcomes. The first criterion was defined as a bad outcome in NMM and a good outcome in NAA and NWMAA. The second criterion was defined as a bad outcome in NMM and NAA and a good outcome in NWMAA. The level of probability was established at *p* < 0.05.

## 3. Results

There was no difference in maternal age, parity, pre-eclampsia, or neuroprotective therapy between the groups. Gestational age at delivery was significantly lower in the NMM group. Fetal lung maturation was rarely found in NMM. Fetal indication for delivery was more often seen in NMM and in NAA than in NWMAA. The majority of cases were delivered by Cesarean section. Neonatal body weight, 5’min Apgar score, pH, and BE were all higher in NWMAA. There was no difference between the groups in term of hospital stay (Table 1). Fetal and maternal indications for delivery are shown in Table 2.

AFI was lower in NMM, while NWMAA had higher BPS. UA PI was lower in NWMAA. Qualitative measurement of UA blood flow also confirmed better blood flow in NWMAA. NWMAA had higher MCA PI and CPR and fewer cases with CPR < 5th percentile. NMM had higher DV PI, and more often had DV Pi > 95th percentile or absent/reversed A wave, and pulsatile blood flow in UV. The incidence of pathological FHRM was higher in NMM and NAA, but the difference was not statistically different (Table 3).

**Figure 1:**
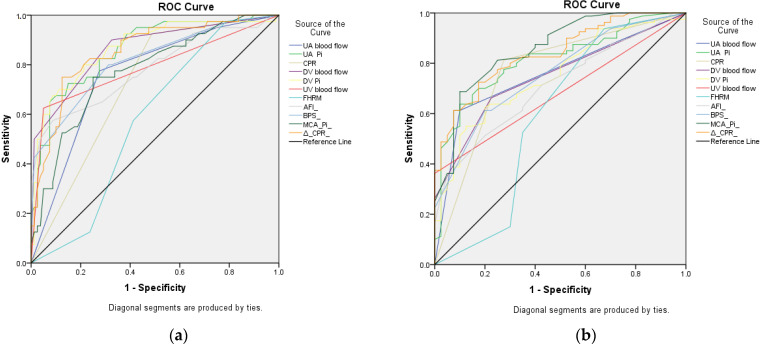
ROC curve for early preterm fetal growth restriction comparing: (**a**) Neonates with morbidity/mortality (bad outcome) with neonates with acidosis/asphyxia but without neonatal morbidity and neonates without neonatal morbidity/acidosis/asphyxia (good outcome); (**b**) Neonates with morbidity/mortality with neonates and acidosis/asphyxia but without neonatal morbidity (bad outcome) with neonates without neonatal morbidity/acidosis/asphyxia (good outcome).

ROC calculated by defining a bad outcome as NMM and a good outcome as NAA and NWMAA, showed the best sensitivity in DV Pi, followed by DV blood flow, UA PI, ΔCPR, BPS, UV blood flow, AFI, MCA PI, UA blood flow and CPR, while FHRM showed no statistical significance (Table 4, Figure 1a).

ROC calculated by defining a bad outcome as NMM and NAA and a good outcome as NWMAA in early FGR showed the best sensitivity in MCA PI, followed by ΔCPR, UA PI, CPR, UA blood flow, BPS, DV Pi, DV blood flow, AFI and UV blood flow, while FHRM showed no statistical significance (Table 4, Figure 1b).

## 4. Discussion

FGR represents one of the leading causes of perinatal morbidity and mortality which is eight times higher in growth-restricted neonates. Morbidities are usually consequences of undeveloped organ functions of the lungs (RDS, bronchopulmonary dysplasia), immune system (sepsis, pneumonia, meningitis), cerebral blood vessels (hemorrhage, leucomalatia) and bowels (NEC). Growth-restricted neonates are at a higher risk of long-term sequels, such as impaired neurological and cognitive development or cardiovascular and metabolic changes in adulthood. FGR is one of the major causes of preterm birth and intrapartum asphyxia [1,2,3]. Based on the prognosis, FGR is classified as early (≤ 32 weeks) or late (>32 weeks). Early FGR has poorer prognosis, but there is also controversy about the timing of delivery [2,3]. Management of placental FGR is based upon gestational age and severity of fetal compromise. Intensive fetal monitoring should help in the determination of timely delivery, although optimized delivery usually does not decrease neonatal and long-term morbidity [6,19].

We chose early preterm FGR for our study because of the number of controversies about the monitoring and timing of delivery. Lower gestational age in NMM implies that extreme prematurity with FGR increases the risk for neonatal morbidity and mortality. Jensen and associates in their study reported increased risk of neonatal morbidity and mortality before 32 weeks in inverse correlation with gestational age [20]. Corticosteroid application occurred more often in NWMAA in early FGR, confirming that fetal lung maturation influences a better outcome, although this may be explained by lower gestational age in the cases where corticosteroid has not been applied. Fetal indications for delivery were seen rarely in NWMAA, suggesting better fetal condition. Similar results were reported by a TRUFFLE study finding better conditions, but slightly insignificantly better survival in cases delivered for maternal indication [21]. The higher 5’min Apgar score, pH, and BE observed in NWMAA, as well as the lower neonatal body weight in NMM, suggest that more severe forms of FGR have a worse outcome.

As oligohydramnios is a consequence of impaired renal function, higher incidence of reduced AFI in NMM may imply worse fetal condition. The incidence of oligohydramnios in FGR is relatively small, and in about 5% of cases is connected to admittance to the NICU, but not with other complications [22,23]. In our study, oligohydramnios (AFI ≤ 50) was rare, but in severe cases amniotic fluid was reduced (AFI < 100).

An earlier study suggests that BPS is the best parameter for making decisions about delivery, with a sensitivity of 82% [24], and so BPS was recommended twice a week in FGR with pathological UA blood flow. Other studies show that circulatory changes precede worsening of BPS, and that fetal death may happen soon after blood flow redistribution [25,26,27]. On the other hand, BPS correlates with fetal pH over 90% and may help in identifying fetuses that should be delivered [26]. Although BPS indicates fetal acidemia, it is not predictable for future fetal well-being. Therefore, an integrated surveillance strategy that includes BPS for monitoring FGR is recommended. Our results show lower BPS in cases with worse outcomes.

We decided to observe UA blood flow by measuring PI and defining groups of normal to pathological blood flow in order to investigate both models of interpretation. We found that UA blood flow was better in NWMAA. Pathological UA blood flow relates to adverse outcome and neurological sequels [28]. Normal UA blood flow is almost never connected with adverse outcomes. The finding of absent/reversed end-diastolic flow in UA indicates severely impaired placental perfusion and adverse outcomes as well. Pathological UA should be interpreted along with other circulatory changes and monitoring should include other Doppler measurements with BPS and FHRM [29,30,31,32]. The highest incidence of pathological UA in NMM confirms the connection between pathological UA blood flow and adverse outcome.

Centralization, i.e., redistribution of blood flow, occurs as a response to chronic hypoxemia and is activated in advanced stages of FGR. The goal of redistribution of blood is to preserve flow through the vital organs—brain, heart, spleen and adrenal gland—at the expense of reducing perfusion to other peripheral organs, thus enabling the survival of the fetus for a significant period. In the brain, vasodilatation occurs via chemoreceptors and baroreceptors and is manifested by a decrease in MCA PI value. With the progression of fetal hypoxemia, the appearance of pathological CPR occurs. Cerebral blood flow reflects changes in the whole circulation and has an essential role in the monitoring of FGR fetuses, with recommendation for serial measurements. Pathological cerebral circulation is connected with adverse outcomes, while connection with neurological sequels is contradictory. The role of CPR in the decision to indicate delivery is relatively small in early FGR, as in early pregnancies fetuses tolerate circulatory changes better. In early FGR, pathological CPR is important in selecting the risk group in which the procedure of intensive monitoring should be applied [31,32,33,34]. We found decreased MCA PI and lower CPR in NMM and NAA. The presence of pathological cerebral blood flow in the NMM and NAA may be explained by adverse outcome in the cases with blood redistribution.

Pathological venous blood flow was registered more often in NMM. Pathological venous blood flow is a result of myocardial constriction representing cardiac dysfunction in terminal changes in chronic hypoxemia and is a sign of severe fetal compromise with increased risk for adverse outcome and late sequels [35,36,37,38,39]. It is an obligatory indication for delivery. The fact that pathological venous blood flow was observed mostly in NMM confirms the value of venous blood flow in the prediction of adverse outcomes.

As fetal heart rate (FHR) is a function of the autonomic nervous system, FHR monitoring enables insight into the condition of the autonomic nervous system. FHRM by conventional cardiotocography (CTG) monitoring represents a standard for prenatal fetal surveillance via visual inspection of basal frequency, long-term variability, accelerations and decelerations, while computerized cardiotocography (cCTG) and monitoring of short-term variability provides numerical results avoiding interobserver and intraobserver variability [38,40,41]. Although cCTG is proven to be superior, the conventional CTG remains a gold standard in monitoring fetal well-being [39]. Fetal heart rate variability decreases as a result of the occurrence of hypoxia, while spontaneous decelerations occur in progressive hypoxia and acidemia. Repeated spontaneous decelerations indicate imminent delivery. Contraction stress tests may allow early intervention before fetal acidemia occurs and helps in the reduction in adverse outcome [42]. We did not find any difference in FHRM monitoring, likely because we intervened before the outcome worsened.

ROC calculated when NMM were compared to the other two groups showed the highest sensitivity for DV blood flow, while ROC calculated when NMM and NAA were compared to NWMAA, showed the highest sensitivity for cerebral blood flow. Monitoring FGR pregnancies presents an enigma concerning diagnostic methods and monitoring interval. Different studies have found different diagnostic parameters as the best predictors of adverse outcome and propose different protocols [26,36,43]. Cochrane database recognizes only UA blood flow as a method for reducing the risk of perinatal death [44]. There is currently no better intervention for the treatment of FGR than delivery, although no study has shown benefit from either intensive monitoring or emergency delivery. False positive pathological Doppler blood flow may indicate unnecessary premature delivery, but fetal death may occur unexpectedly due to unrecognized fetal compromise or inadequate monitoring interval, so in early preterm FGR balance should be made between severe prematurity and the risk of fetal death. Because in placental FGR adverse outcome is a consequence of chronic malnutrition and hypoxemia, monitoring should pay attention to revealing chronic hypoxemia, such as Doppler in umbilical, cerebral, and venous circulation. There are studies suggesting optimal protocol for monitoring FGR, especially early preterm pregnancies using different models, distinguishing DV blood flow, cCTG and BPS [21,24,40,45,46]. A scoring system incorporating both BPS and Doppler parameters was proposed, but with modest sensitivity and specificity [45].

Tests in early FGR showed higher sensitivity and the majority of them have good to moderate success. The difference in test values calculated by two criteria highlights the significance of different tests in prediction of good or bad outcomes. In early FGR, if there is pathological DV blood flow, a bad outcome may be expected, while in normal cerebral blood flow a good outcome may be expected. As none of the diagnostic tests have shown excellent sensitivity/specificity, a model of simultaneous use of all diagnostic methods is the only choice.

## 5. Conclusions

We may conclude that expectant management with intensive fetal surveillance is the best choice in the cases of fetal growth restriction before 32 weeks. Different diagnostic parameters are useful for monitoring early fetal growth restriction. In early fetal growth restriction, normal cerebral blood flow strongly predicts a good outcome, while pathological venous blood flow is associated with bad outcomes. Despite established monitoring protocols, in fetal growth restriction before 32 weeks, individualized management remains the best option for the optimal timing of delivery.

## Figures and Tables

**Table 1:** Patient characteristics in early preterm growth-restricted neonates (≤32 wg).

	NMM N = 40	NAA N = 40	NWMAA N = 40	*p*	Overall N = 120
Maternal age	31.3 ± 6.4	32.6 ± 5.7	30.9 ± 6.6	0.456 ^b^	31.6 ± 6.2
Gestational age (weeks)	28.94 ± 1.87	30.46 ± 1.08	30.91 ± 1.0	<0.001 ^b^	30.1 ± 1.6
Parity, *n* (%) 1	30 (75)	26 (65)	25 (62.5)	0.548 ^a^	81 (67.5)
2	5 (12.5)	10 (25)	11 (25)		26 (21.7)
3+	5 (12.5)	4 (10)	4 (10)		13 (10.8)
Pre-eclampsia, *n* (%)	28 (70)	29 (72.5)	31 (77.5)	0.742 ^a^	88 (73.3)
Corticosteroid, *n* (%)	32 (80)	38 (95)	40 (100)	0.005 ^a^	110 (91.7)
Neuroprotection—MgSO_4_, *n* (%)	19 (47.5)	25 (62.5)	25 (62.5)	0.100 ^a^	69 (57.5)
Fetal indication for delivery, *n* (%)	31 (77.5)	20 (50)	14 (35)	0.001 ^a^	65 (53.7)
Cesarean Section, *n* (%)	40 (100)	40 (100)	39 (97.5)	0.365 ^a^	119 (99.7)
N gender, *n* (%) Male	22 (55)	18 (45)	23 (57.5)	0.496 ^a^	63 (52,5)
Female	18 (45)	22 (55)	17 (42.5)		57 (47.5)
Neonatal body weight (g)	907.3 ± 146.7	1284.3 ± 166.9	1448.5 ± 166.2	<0.001 ^b^	1213.3 ± 277.5
5’Apgar score	5.53 ± 1.77	7.15 ± 0.97	8.1 ± 0.81	<0.001 ^b^	6.93 ± 1.64
pH	7.13 ± 0.13	7.17 ± 0.44	7.29 ± 0.04	<0.001 ^b^	7.19 ± 0.11
Base excess	8.15 ± 5.14	7.63 ±3.09	2.52 ±1.11	0.007 ^b^	6.10 ±4.33
Hospital stay (days)	66.78 ± 42.4	58.2 ± 17.7	55.9 ± 18.9	0.208 ^b^	60.3 ± 28.8

Abbreviations: NMM—Neonatal morbidity/mortality; NAA—Neonatal acidosis/asphyxia; NWMAA—Neonates without neonatal morbidity/acidosis/asphyxia; ^a^ χ^2^—chi-square test; ^b^ ANOVA test Test; *p* = statistical significance.

**Table 2:** Indications for delivery.

	NMM N = 40	NAA N = 40	NWMAA N = 40	Overall N = 120
Fetal indication for delivery, *n* (%)	31 (77.5)	20 (50)	14 (35)	65 (54.2)
FHRM (Decelerations/positive CST)	14	9	7	30
BPS changes/Olygohydramnio	7	5	3	15
Doppler changes	10	6	4	20
Maternal indication for delivery, *n* (%)		20 (50)	16 (65)	55 (45.8)
HELLP	3	4	8	15
Severe pre-eclampsia	4	11	13	28
Superimposed pre-eclampsia	2	5	5	12

Abbreviations: NMM—Neonatal morbidity/mortality; NAA—Neonatal acidosis/asphyxia; NWMAA—Neonates without neonatal morbidity/acidosis/asphyxia; FHRM—Fetal heart rate monitoring; CST—Contraction Stress Test; BPS—Biophysical Profile Score; HELLP (Hemolysis, Elevated liver enzymes, Low Platelet).

**Table 3:** Diagnostic parameters in early preterm growth-restricted neonates (≤32 wg).

	NMM N = 40	NAA N = 40	NWMAA N = 40	*p*	Overall N = 120
AFI	74.48 ± 30.26	100.63 ± 25.65	110.38 ± 26.42	<0.001 ^b^	95.16 ± 31.24
BPS	5.45 ± 1.87	7.30 ± 1.40	8.18 ± 1.43	<0.001 ^b^	6.98 ± 1.94
UA PI	1.78 ± 0.22	1.52 ± 0.26	1.32 ± 0.28	<0.001 ^b^	1.54 ± 0.32
UA blood flow, *n* (%)				<0.001 ^a^	
PI ≤ 95th percentile	2 (5)	8 (20)	12 (30)		22 (18.3)
PI > 95th percentile	7 (17.5)	14 (35)	24 (60)		45 (37.5)
AREDV	31 (77.5)	18 (45)	4 (10)		53 (44.2)
MCA Pi	1.24 ± 0.16	1.32 ± 0.14	1.54 ± 0.2	<0.001 ^b^	1.37 ± 0.21
CPR	0.72 ± 0.19	0.92 ± 0.24	1.25 ± 0.41	<0.001 ^b^	0.96 ± 0.36
ΔCPR, *n* (%)				<0.001 ^a^	
≥5th percentile	3 (7.5)	12 (30)	29 (72.5)		44 (36.7)
<5th percentile	37 (92.5)	28 (70)	11 (27.5)		76 (63.3)
DV PI	1.10 ± 0.42	0.64 ± 0.3	0.55 ± 0.22	<0.001 ^b^	0.76 ± 0.4
DV blood flow, *n* (%)				<0.001 ^a^	
PI ≤ 95th percentile	4 (10	23 (57.5)	31 (77.5)		58 (48.3)
PI > 95th percentile	16 (40)	16 (40)	9 (22.5)		41 (34.2)
ARA wave	20 (50)	1 (2.5)	0 (0)		21 (17.5)
UV blood flow, *n* (%)				<0.001 ^a^	
Laminar	15 (37.5)	36 (90)	40 (100)		91 (75.8)
Pulsatile	25 (62.5)	4 (10)	0 (0)		29 (24.2)
FHRM, *n* (%)				0.130 ^a^	
Normal/Silent, *n* (%)	17 (42.5)	21 (52.5)	26 (65)		64 (53.3)
Spontaneous decelerations/Positive CST, *n* (%)	23 (57.5)	19 (47.5)	14 (35)		56 (46.7)

Abbreviations: NMM—Neonatal morbidity/mortality; NAA—Neonatal acidosis/asphyxia; NWMAA—Neonates without neonatal morbidity/acidosis/asphyxia; AFI—Amniotic fluid index; BPS—Biophysical profile score; UA—Umbilical artery; PI—Pulsatility index; UA blood flow—Umbilical artery blood flow; MCA—Middle cerebral artery; CPR—Cerebral-Placental ratio numeric value; ΔCPR—Cerebral-Placental ratio qualitative assessment; DV—Ductus venosus; DV blood flow—Ductus venosus blood flow; UV umbilical vein; FHRM—Fetal heart rate monitoring; CST—Contraction Stress Test; ^a^ χ^2^—chi-square test; ^b^ ANOVA test *t* Test; *p* = statistical significance.

**Table 4:** Diagnostic parameter sensitivity and specificity—ROC by comparing: I—neonates with morbidity/mortality (bad outcome) with neonates with acidosis/asphyxia but without neonatal morbidity and neonates without neonatal morbidity/acidosis/asphyxia (good outcome) and II—neonates with morbidity/mortality with neonates and acidosis/asphyxia but without neonatal morbidity (bad outcome) with neonates without neonatal morbidity/acidosis/asphyxia (good outcome).

Test Result Variable(s)	I		II	
	AUC (Asymptotic 95% CI)	*p*	AUC (Asymptotic 95% CI)	*p*
AFI	0.783 (0.688–0.877)	<0.001	0.711 (0.619–0.803)	<0.001
BPS	0.816 (0.732–0.900)	<0.001	0.758 (0.671–0.845)	<0.001
UA PI	0.863 (0.793–0.932)	<0.001	0.814 (0.736–0.891)	<0.001
UA blood flow	0.760 (0.669–0.851)	<0.001	0.758 (0.670–0.846)	<0.001
MCA PI	0.774 (0.686–0.863)	<0.001	0.859 (0.789–0.928)	<0.001
CPR	0.719 (0.627–0.810)	<0.001	0.769 (0.674–0.864)	<0.001
ΔCPR	0.855 (0.783–0.927)	<0.001	0.840 (0.770–0.910)	<0.001
DV PI	0.869 (0.798–0/940)	<0.001	0.750 (0.662/0.839)	<0.001
DV blood flow	0.863 (0.790–0.936)	<0.001	0.748 (0.662–0.835)	<0.001
UV blood flow	0.788 (0.690–0.885)	<0.001	0.681 (0.588–0.774)	<0.001
FHRM	0.577 (0.475–0.678)	0.173	0.592 (0.468–0.716)	0.102

Abbreviations: CI—Confidence Interval; AUC = Area Under the Curve; *p* = statistical significance; AFI—Amniotic fluid index; BPS—Biophysical profile score; UA PI—Umbilical artery Pulsatility index; UA blood flow—Umbilical artery blood flow; MCA PI—Middle cerebral artery; CPR—Cerebral–Placental ratio numeric value; ΔCPR—Cerebral–Placental ratio qualitative assessment; DV PI—Ductus venosus Pulsatility index; DV blood flow—Ductus venosus blood flow; FHRM—Fetal Heart Rate Monitoring.

## Data Availability

The data presented in this study are available on request from the corresponding author. The data are not publicly available due to ethical regulations.
